# Application of Aptamers in Virus Detection and Antiviral Therapy

**DOI:** 10.3389/fmicb.2019.01462

**Published:** 2019-07-03

**Authors:** Xinran Zou, Jing Wu, Jiaqi Gu, Li Shen, Lingxiang Mao

**Affiliations:** ^1^Department of Laboratory Medicine, The Affiliated People's Hospital, Jiangsu University, Zhenjiang, China; ^2^Jiangsu Key Laboratory of Laboratory Medicine, Department of Immunology, School of Medicine, Jiangsu University, Zhenjiang, China; ^3^Zhenjiang Center for Disease Control and Prevention, Jiangsu, China

**Keywords:** aptamers, SELEX, aptasensors, virus detection, antiviral therapy

## Abstract

Viral infections can cause serious diseases for humans and animals. Accurate and early detection of viruses is often crucial for clinical diagnosis and therapy. Aptamers are mostly single-stranded nucleotide sequences that are artificially synthesized by an *in vitro* technology known as the Systematic Evolution of Ligands by Exponential Enrichment (SELEX). Similar to antibodies, aptamers bind specifically to their targets. However, compared with antibody, aptamers are easy to synthesize and modify and can bind to a broad range of targets. Thus, aptamers are promising for detecting viruses and treating viral infections. In this review, we briefly introduce aptamer-based biosensors (aptasensors) and describe their applications in rapid detection of viruses and as antiviral agents in treating infections. We summarize available data about the use of aptamers to detect and inhibit viruses. Furthermore, for the first time, we list aptamers specific to different viruses that have been screened out but have not yet been used for detecting viruses or treating viral infections. Finally, we analyze barriers and developing perspectives in the application of aptamer-based virus detection and therapeutics.

## Introduction

Aptamers are small single-stranded artificial nucleotides (DNA or RNA), in the range of 10–100 nucleotides (nt), that have a remarkable ability to bind to their targets. Aptamer targets include a variety of small molecules such as amino acids, nucleotides, and antibiotics (Ellington and Szostak, 1992), but can also be larger, including proteins (Schneider et al., 1992), viruses and bacteria (Torres-Chavolla and Alocilja, 2009) as well as other cells (Ku et al., 2015). The secondary and tertiary structures of aptamers ensure the binding specificity to their targets via aptamer-target recognition, and may involve aromatic rings, π-π system stacking, van der Waals forces, electrostatic interactions or hydrogen bonding (Szpechcinski and Grzanka, 2006; Ku et al., 2015). Because of their binding specificity to their targets, aptamers are often compared to antibodies and are also known as chemical antibodies or artificial antibodies (Banerjee, 2010; Wang et al., 2016).

**Figure 1:**
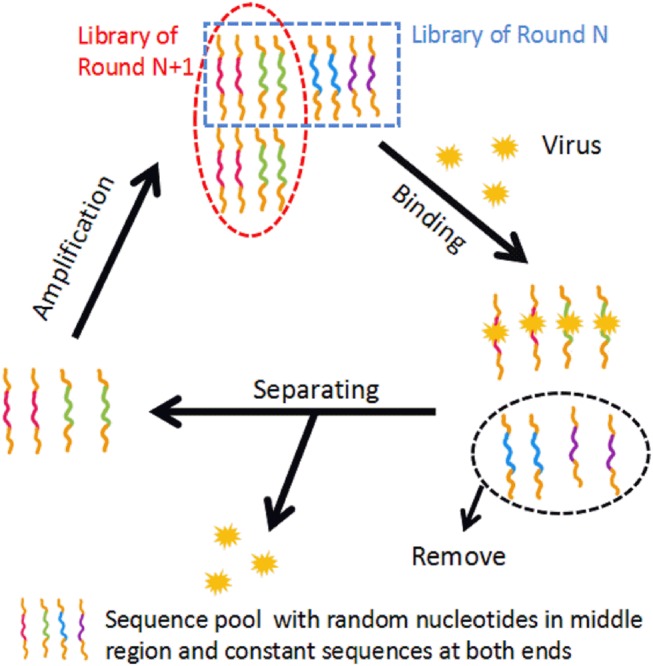
The basic mechanism of conventional SELEX. In the binding step, the sequence pool is incubated with the target. In the separating step, unbound sequences are removed and bound sequences are separated from the target. In the amplification step, the separated sequences are amplified, building a new sequence pool for the next iteration of SELEX.

The selection method of aptamers, Systematic Evolution of Ligands by Exponential Enrichment (SELEX), is an *in vitro* process. Briefly, SELEX is based on iterative cycles of binding, separating and amplification of nucleotides. The basic mechanism of SELEX is shown in Figure 1. The first step of conventional SELEX is to incubate the sequence pool with the target (protein, nucleic acid, etc.). The sequence pool is a nucleic acid library containing 10^14^-10^15^ variants of random 30–100 nucleotides flanked by constant sequences at both ends. The random region contains the sequences that will be tested for high specificity and affinity to the target. Second, sequences that bind the target is kept, while unbound nucleotides are removed. The third step is to purify and amplify the bound sequences to form a new sequence pool for the next cycle. This cyclic process is typically repeated 8–15 times before achieving the desired aptamer sequence pool (Torres-Chavolla and Alocilja, 2009; Davydova et al., 2016). A negative selection, or counter selection, step involves incubating the sequence pool with target analogs, undesired subtypes, or the unbound sequences. This step can take place before or after target incubation to improve the specificity of candidates (Haller and Sarnow, 1997; Iwagawa et al., 2012). Biotechnology companies provide aptamer-related services, including the construction of sequence pools, aptamer selection, aptamer synthesis, and aptamer modification.

Currently, viral infection is a serious threat for human beings. Although antibody-based detection methods and drugs are widely used in clinics, their popularity is hindered by high cost, antibody instability and the limitation of target types (Resch, 2017; Seo and Gu, 2017). A comparison between aptamers and antibodies is shown in Table 1. Aptamers have great potential as a feasible tool in virus detection and therapeutics.

**Table 1:** Comparative properties of aptamers and antibodies.

**Property**	**Aptamer**	**Antibody**	**References**
Time needed for selection	Several weeks	Several months	-
Cost of the selection	~$4000 for individual aptamer sequences	~$8,000 for mouse monoclonal antibody ~$20,000 for rabbit monoclonal antibody	-
Synthesis and manufacture	Chemically synthesis *in vitro*	Produced in animal and then by recombinant methods	-
Modification	Easy and controllable	Limited and uncontrollable	Binning et al., 2012; Wandtke et al., 2015
Stability	Stable in different environmental conditions	Requiring special conditions for the storage and handling	Davydova et al., 2016; Wang et al., 2016
Batch-to-batch variation	Little or no	Difficult to avoid	Torres-Chavolla and Alocilja, 2009; Davydova et al., 2016
Size	5–25 kDa	Usually more than 125 kDa	Banerjee, 2010
Chemical property	Mainly nucleic acids	Protein	-
Target range	Wide to almost anything	Limited to antigenic targets	O'Sullivan, 2002; Proske et al., 2005; Torres-Chavolla and Alocilja, 2009; Wandtke et al., 2015; Davydova et al., 2016
*In vivo* complications	No intrinsic immune response	May lead immune response	Szpechcinski and Grzanka, 2006
Specificity and affinity	High	High	-
Clinical application	Immature	Mature	-

## Applications of Aptamers In Virus Detection

Current techniques to diagnose viral infections include virus isolation in tissue cultures, immunological and molecular methods. However, these methods have a variety of limitations; for example, they are technically demanding, costly and can produce false positive or false negative results, whereas aptamer-based assay for virus detection may improve these drawbacks to some extent (Li et al., 2016; Vidic et al., 2017).

A biosensor is an analytical device that combines a bioreceptor and a transducer. The bioreceptor recognizes and binds the target with high sensitivity and selectivity, and averts interference from other microorganisms or molecules (Hong et al., 2012). The transducer then translates and outputs biological signals from the interaction between the analyte and the bioreceptor (Han et al., 2010). Aptamer-based biosensors, also called aptasensors, use aptamer as bioreceptors (also named capturing aptamer/probe) or transducers (also named signal aptamer/probe) (Cheng et al., 2009; Hianik et al., 2009). Aptasensors are mainly classified into optical and electronic aptasensors based on the type of transducer.

### Optical Aptasensors

Optical aptasensors for virus detection can be classified into six categories based on the optical principles used for material detection. These categories are surface plasmon resonance (SPR) aptasensors, colorimetric aptasensors, chemiluminescence (CL) aptasensors, fluorescence aptasensors, surface-enhanced Raman scattering (SERS) aptasensors, and interferometry aptasensors.

#### SPR-Based Aptasensors

**Figure 2:**
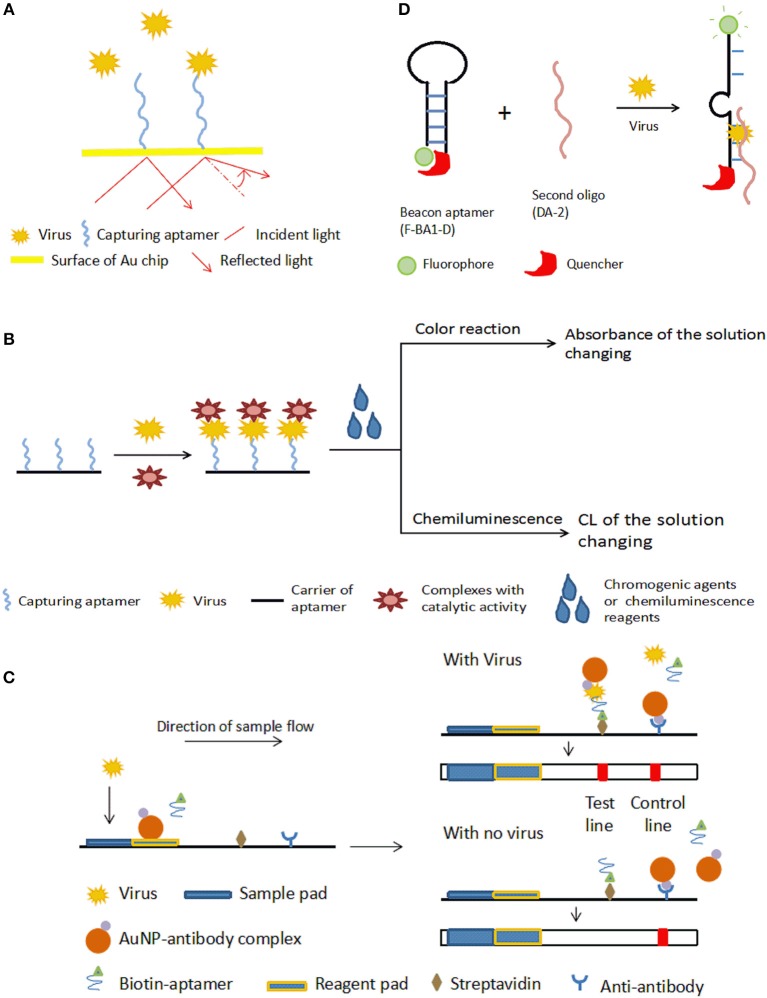
Schematic illustration of optical aptasensors. **(A)** Mechanism of SPR aptasensors. The aptamer-virus interaction changes the angle of reflected light, which indicates the amount of virus captured by the aptamers. **(B)** Mechanism of typical colorimetric-based aptasensor and CL aptamers. The aptamer is incubated with the virus, then catalytic-active complexes that bind the captured virus are added. Appropriate chromogenic or CL reagents are added to affect the color or luminous intensity of the sample. The change in color or luminous intensity is correlated to the amount of virus in the sample. **(C)** Mechanism of LFA-based aptasensor. In the presence of the target virus, both the aptamer and the AuNP-antibody complex bind to the virus, and the biotin on the aptamer enables the complex to be bound onto the streptavidin on the test line, allowing detection by the color of GNPs. The color of the test line does not change if there is no target in the sample. With or without virus, the AuNP-antibody complex is caught by the anti-antibody on the control line to cause a color change as a control. **(D)** Mechanism of the fluorescent quench method. Target virus can bind with F-BA1-D and DA-2 and change the structure of F-BA1-D, separating the fluorophore and the quencher and releasing fluorescence.

SPR measures the resonance of free electrons in some metal films by measuring the change of refractivity of the material bound on a surface (Adamczyk et al., 1999). For a typical SPR aptasensor, the capturing aptamer is immobilized on a metal surface, most often gold. The binding between viruses and aptamers changes the thickness of the gold surface, and as a result, the refractive index varies. The bound target on the surface can be quantified by monitoring the angles or intensity of the polarized light (Nguyen et al., 2015). The principle of SPR aptasensors is shown in Figure 2A. SPR sensors have certain advantages, including that no marking is required, miniaturization and automation (Skottrup et al., 2008).

Bai et al. (2012) developed an SPR aptasensor for quickly detecting avian influenza virus (AIV) H5N1 within 1.5 h, with a detection range from 0.128 to 1.28 hemagglutination units (HAUs). Compared with other detection methods, this aptasensor was fast and portable, but the sensitivity was inferior to virus isolation and PCR methods. Similarly, Tombelli et al. (2005) proposed an SPR aptasensor for detecting the HIV-1 Tat protein. In another study by Nguyen et al. (2016), a pair of aptamers, IF10 and IF22, bound different sites of the same H5N1 virus, acting as the capturing probe and signal probe, respectively. This built a sandwich-type SPR biosensor platform for the sensitive detection of H5N1 viruses. In this aptasensor, the H5N1 virus was first bound by biotin-labeled aptamer IF10, which was fixed on the surface of the streptavidin-coated SPR gold chips. Then, the report aptamer IF22 linked with gold nanoparticles (AuNPs) combined with the virus captured on the SPR chips, and the AuNPs on IF22 enhanced the angle shift. By amplifying the signal with the sandwich system, the detection sensitivity of this biosensor was found to be 200 EID50/ml (50% embryo infective dose/ml) for H5N1 virus in feces samples, comparable with the sensitivity of ELISA.

#### Colorimetric-Based Aptasensors

In colorimetric detection, a shift of color is measured, which is either directly observed by eye or using a spectrophotometer. Colorimetric methods have merits, such as their low cost, simplicity, and portability, and thus have been widely applied in aptasensors (Feng et al., 2014; Ng et al., 2016). The principle of typical colorimetric-based aptasensors is shown in Figure 2B.

##### Nanomaterial-assisted colorimetric aptasensors

For this type of aptasensor, nanomaterials support the capturing aptamer, and some also take part in the signal conversion. To fabricate an aptasensor for detecting the influenza A virus, Chen et al. (2016) used an H3N2-specific aptamer modified with magnetic beads to capture the virus. AuNPs were modified with glucose oxidase (GOx) and concanavalin A (Con A), and these Con A-GOx-AuNP complexes were used for the output signal. The complexes bound the virus through a Con A-glycan interaction, and the GOx transformed the chemical signal into a color signal. This aptasensor detected the H3N2 virus at levels as low as 11.16 μg/ml with the help of a UV-vis instrument.

The hydrothermal reaction of HAuCl_4_ and graphene oxide produces graphene/AuNPs (Wang et al., 2010). In addition, the graphene/AuNPs have a peroxidase-like activity, mediating a catalytic reaction that is accompanied by a color change (He et al., 2011; Liang et al., 2011). Based on the graphene/AuNP hybrids, Liu et al. (2012) proposed a label-free aptasensor for detecting hepatitis C virus (HCV). In this system, the ssDNA aptamer prevents the peroxidase substrates from contacting the active interface and depresses the catalytic ability of the graphene/AuNPs. However, catalytic activity is recovered when viruses are present because the combination of the aptamer and virus reduces this catalytic hindrance. Finally, the substrate 3,3′,5,5′-tetramethylbenzidine is added to the system to visualize the result. The resulting color changes are highly correlated to the amount of virus.

##### Enzyme-linked aptamer assays (ELAA)

An ELISA is a basic diagnostic method for detecting complex target molecules. In ELAA, aptamers are used as a substitution for antibodies as the bio-receptor or the transducer (Nie et al., 2013). ELAA is also known as an enzyme-linked oligonucleotide adsorption test (ELOSA or ELONA) or an enzyme-linked aptasorbent assay (ELASA) (Rasoulinejad et al., 2016; Stoltenburg et al., 2016).

An ELAA for detecting the influenza A virus H5N1 used the aptamer RHA0006, which targets the hemagglutinin (HA) protein (Shiratori et al., 2014). In this aptasensor, the aptamer was immobilized on wells to capture the HA protein, and another 3′-biotinylated aptamer induced a color reaction in cooperation with streptavidin (SA)-horseradish peroxidase and the chromogen reagent 3,3′,5,5′-tetramethylbenzidine. This sandwich enzyme-linked aptasensor also recognized the H1N1 and H3N2 subtypes. The lower limit of detection reached 0.1 μg/well. Analogous ELAAs have been used to detect human norovirus (Escudero-Abarca et al., 2014), Zika virus (Lee and Zeng, 2017), and HCV (Park et al., 2013). In developing the Zika ELAA, researchers tested different pairs of capturing agent and detection agent. The aptamer/antibody pair exhibited the best detection, comparable to capacitive or impedimetric immunoassays and antibody-based ELISA kits. The detection limit of the aptamer1/aptamer2 pair was worse than the aptamer/antibody pair, but the author postulated that further research to optimize the aptamer/antibody pair may improve the detection effect. The aptamer has lower production cost and displayed a high degree of batch-to-batch consistency (Lee and Zeng, 2017).

##### Aptasensors based on lateral flow assay (LFA)

A lateral flow immunochromatographic assay (LFA) takes advantage of a series of capillary beds that transport fluid. LFA is widely used in clinical point-of-care detection, such as detecting levels of human chorionic gonadotrophin, HIV, HBV, and so on. Based on LFA, Le et al., 2017 put forward a method for detecting a multiplex strain-specific influenza virus. In this aptasensor, the virus was added into the sample pad and was conjugated with a biotinylated aptamer and an AuNP-labeled monoclonal antibody to form a complex at the conjugate pad. When the fluid reached the text line where SA was located, the conjugate was bound by biotin-SA, leading to a visible color change in the text line. The detection limit was about 2 × 10^6^ virus particles. The working mechanism of aptamer-based LFA is shown in Figure 2C.

#### CL Aptasensors

CL is defined as material molecules generating optical radiation after absorbing chemical energy. In CL methods, the intensity of the luminous radiation reflects the concentration of the analytes. CL analysis has high sensitivity (detection limit of 10^−12^ to 10^−21^ mol) due to the ability to carry out photon metering without interference from scattered light background when an external excitation source exists, as well as a wide linear range (3–6 orders of magnitude). CL assays are another technology extensively applied in clinical diagnosis. The detecting principle of a typical CL aptasensor is similar to the principle of colorimetric-based aptasensors, shown in Figure 2B.

Based on a CL immunosorbent assay, Ahn et al. (2009) developed an aptasensor to detect severe acute respiratory syndrome coronavirus (SARS-CoV), with an aptamer capturing the SARS-CoV N protein. An enzyme-labeled secondary antibody to the N protein was employed to transduce the signal. This aptasensor detected SARS-CoV N protein at levels as low as 2 pg/ml. According to an analogous principle, Xi et al. (2015) constructed an aptasensor for detecting hepatitis B surface antigen (HBsAg). In this aptasensor, Fe_3_O_4_-SiO_2_ magnetic NPs were connected with the aptamer to help separate the targets from the sample. The linear range of this aptasensor was 1–200 ng/ml. This aptasensor had a lower detection limit than the limit of the ELISA used in clinical applications.

#### Fluorescence Aptasensors

Fluorescent aptamer biosensors use fluorophores as the signal output element. The outcome is reflected by changes in the fluorescence intensity or by the production of fluorescence polarization (Dwivedi et al., 2010; Ohk et al., 2010).

##### Aptasensor response with fluorescence intensity

Wang et al. (2016) applied a fluorescent-labeled universal aptamer to build an integrated microfluidic detection device for multivirus diagnosis. In this aptasensor, aptamers distinguished influenza A H1N1, H3N2, and influenza B viruses. For this aptasensor, an aptamer was modified on magnetic beads to catch the virus, and another fluorescence-labeled universal aptamer marked the captured analyte. Detection could be finished in 20 min, enabling point-of-care identification of influenza infection. In another study, a sol-gel protein chip was generated for detecting HCV core antigen in patient serum. In this chip, the aptamer was used to capture the virus, and anti-HCV and Cy3-labeled goat secondary antibodies were applied as signal probes (Lee et al., 2007).

Hmila described an aptamer-real-time-PCR method to detect the H9N2 influenza virus (Hmila et al., 2017). The capturing aptamer, specific to H9N2, was attached onto a particular strip. After virus binding by the capturing aptamer, a reporter aptamer was added into the system to bind the virus. The content of virus was calculated by measuring the bound reporter aptamer using the TaqMan RT-PCR reaction. This PCR method directly used swab samples without extracting nucleic acids, yielding a limit 1000-fold lower than a clinical ELISA. Liu et al. (2019) designed an aptamer selection strategy and identified two candidates for human noroviruses. These aptamers successfully detected human noroviruses from clinical samples as part of an *in situ* capture RT-qPCR assay.

In 2000, Yamamoto and his team reported a detection method to analyze the Tat protein of HIV-1 using aptamer-derived oligomers (Yamamoto et al., 2000a). They selected an aptamer RNA^Tat^ specific to the HIV Tat protein (Yamamoto et al., 2000b). To build a molecular beacon aptamer, the aptamer RNA^Tat^ was split into two oligomers. The beacon aptamer, named F-BA1-D, had a hairpin structure in its body region, a fluorophore at the 5′-end, and a quencher at the 3′-end. The hairpin structure placed the quencher and fluorophore near each other, inhibiting fluorophore emission. The other oligomer, DA-2, was a non-structured oligomer. As shown in Figure 2D, when the HIV-1 Tat protein was present, a stabilized ternary complex (Tat/F-BA1-D/DA-2) formed, in which the fluorophore and the quencher were separated, and fluorescent light was released (Yamamoto et al., 2000a). In Xiao's research, an aptamer specific to the prion protein PrP^C^ similar to the aptamer RNA^Tat^ mentioned above was designed. The detection range of this fluorescence aptamer sensor was 1.1-44.7 g/l, and the minimum limit of detection was 0.3 g/l (Xiao et al., 2009). This fluorescence quench assay was used to detect Influenza A virus DNA and the dengue virus genome (Fletcher et al., 2010; Liu et al., 2017).

Metal-enhanced fluorescence occurs when the emission of the fluorophore is enhanced around specific metal materials, modifying spectral characteristics and reducing photophysical constraints. Pang et al. (2015) applied metal-enhanced fluorescence to design an aptasensor for detecting H5N1. The main reagents included a core–shell of Ag-SiO_2_ NPs, aptamers and thiazole orange. When the H5N1 or HA protein was captured by the aptamers, the conformation of the aptamers changed into a G-quadruplex structure, causing thiazole orange fluorescence. This aptasensor detected H5N1 in both aqueous solution and patient serum. The detection process could be completed in under 30 min.

Utilizing the chemiluminescent resonance transfer strategy, Kim et al. (2018) designed an aptasensor for detecting norovirus GII. In this aptasensor, guanine-modified DNA aptamers were used to capture the target. In the presence of tetra-n-propylammonium hydroxide and dimethylformamide, the guanine of single-stranded DNA reacted with 3,4,5-trimethoxylphenylglyoxal, producing a high-energy intermediate. This intermediate then delivered energy to fluorescent dye (e.g., fluorescein, 6-FAM), which in turn emitted detectable light. The detection limit was 80 ng/ml in tap water.

##### Aptasensor response with fluorescence polarization

Szakács et al. (2018) proposed an aptamer-based fluorescent NP tracking analysis of viruses. In this study, human respiratory syncytial virus (RSV) was the analyte. The fluorescent aptamer bound viral glycoproteins to mark RSV. RSV could then be identified and counted using fluorescent nanoparticle tracking analysis. This analysis method was able to detect viruses larger than ~80–100 nm.

Quantum dots (QDs), also referred to as artificial atoms, are spherical-like inorganic semiconductor fluorescent nanocrystals. Compared with traditional organic dyes, QDs as fluorophores have good stability, perform well in multi-signal detection, and have other advantages as well (Michalet et al., 2005; Ikanovic et al., 2007). QDs are extensively used in fluorescent detection. Based on fluorescence polarization technology, Zhang et al. (2013) utilized a bifunctional DNA aptamer and QDs to develop an aptasensor for detecting H1N1. Briefly, a DNA sequence specific to H1N1 was modified on QDs to build a capturing probe. Another aptamer, specific to both the H1N1 protein and SA, amplified the fluorescence polarization value. This aptasensor detected H1N1 at levels as low as 3.45 nM.

#### SERS-Based Aptasensors

Raman spectroscopy is a type of scattered spectrum that provides “the unique chemical dactylogram” of molecules. When a laser light penetrates the medium, photons collide with the molecule, allowing the interaction of photons and the molecular vibrational energy or rotational energy. The energy of the photons can be adjusted, and the resulting energy changes reveal characteristics about the medium (Sassolas et al., 2011). However, its low sensitivity limits the application of Raman scattering. SERS overcomes this weak point by adsorbing molecules on rough metal surfaces or nanostructures (Otto, 1991; Kneipp et al., 1999; Sassolas et al., 2011; Xu et al., 2013).

Negri et al. (2012) developed a label-free SERS-based aptasensor to detect the viral nucleoprotein of influenza. In the system, Ag nanorods acted as the active substrates, and polyvalent anti-influenza aptamers were immobilized on the surface. The binding of the target and aptamer changed the nucleotide secondary structure, which was sensed by SERS.

#### Interferometry Aptasensors and Other Optical Aptasensors

Interferometry is a label-free technique that measures light intensity generated by the interference of different light beams. The information includes the index of refraction or physical properties, for example, the thickness of a film (Roh et al., 2011; Shah and Duncan, 2014). Roh et al. (2011) detected HCV with an Octet optical platform, where the HCV-specific RNA aptamer was coated on the optical organic film layer of a tip by biotin-SA binding. When the virus attached to the aptamer, the thickness of the organic film changed, and as a result, the signal spectrum changed. This platform had a detection limit of 700 pg/ml.

### Electrical Aptasensors

Electrical aptasensors detect targets as the binding between the aptamer and target causes or changes an electrical signal. These aptasensors are classified as electrochemical aptasensors or piezoelectric transducers based on their detection mechanism.

#### Electrochemical Aptasensors

Typical electrochemical aptasensors immobilize the capturing aptamer on the electrode. Electrochemical aptasensors are categorized based on their method of producing electrical signals. First, in aptasensors without enzymes, the binding of aptamers to targets directly leads to an electrical signal change. Second, in aptasensors with enzymes, the electrical signal change is aided by enzyme catalysis. The third is based on a field-effect transistor (FET).

##### Aptasensors without enzymes

**Figure 3:**
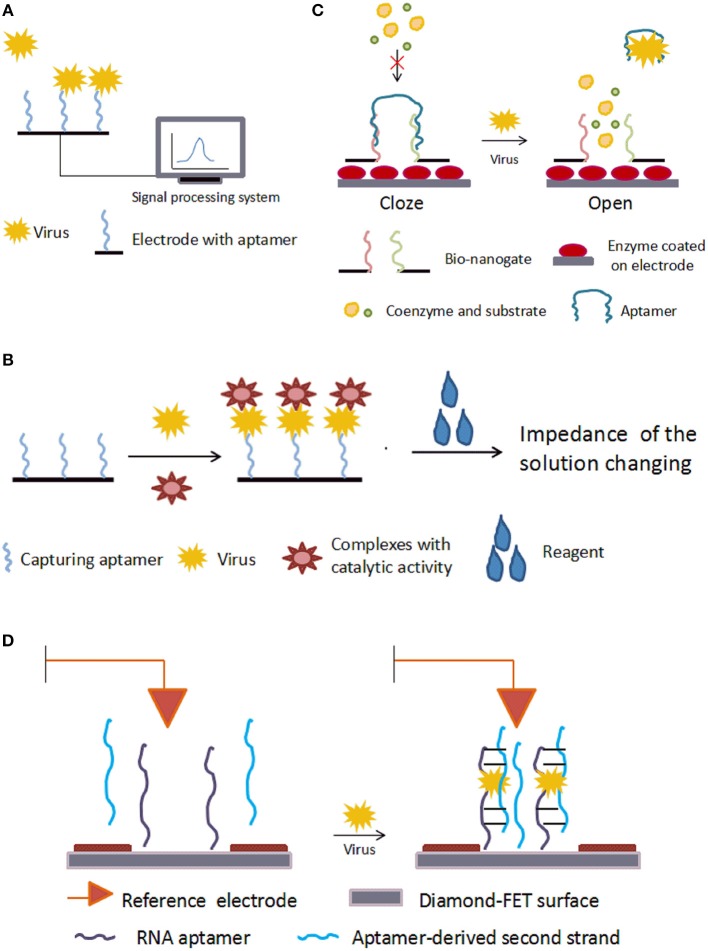
Schematic illustration of electrical aptasensors. **(A)** Mechanism of typical electrochemical aptasensor without enzymes. The combination of aptamer and target virus changes the electrical signal on the electrode. **(B)** Mechanism of electrochemical aptasensor with enzymes. **(C)** Mechanism of a nanogate. With no target virus, the aptamer binds the bio-nanogate to close the “door” and keep the enzyme away from the coenzyme and substrate. The target virus can grab the aptamer from the nanogate, and the enzyme on the electrode reacts with the coenzyme and substrate, leading to a change in the electrical signal on the electrode. **(D)** Mechanism of a diamond-aptamer FET sensor. The aptamer probe is on the diamond-FET surface. In the presence of virus, the aptamer captures the virus and forms a complex with a second aptamer strand. This causes changes in the electric charges on the surface, which is sensed by the electrode.

In aptasensors without enzymes, aptamers are immobilized on the electrode and the binding of aptamer to its target changes the impedance directly. The basic mechanism is shown in Figure 3A. Using microfluidic chips, Lum and his team built an impedance aptasensor to detect AIV H5N1 (Lum et al., 2015). In this biosensor, an SA-covered gold microelectrode was embedded on a microfluidic biochip. The chip was connected to the test-sense and the counter-reference probes of the impedance analyzer. Biotin-modified DNA aptamers were immobilized on the electrode. When the virus sample flowed through the microfluidic module, the aptamer captured the target, increasing the impedance. Detection took under 30 min, and the minimum detection limitation was 0.0128 HAU. Following the same principle, aptasensors were developed to detect influenza A virus (Kirkegaard and Rozlosnik, 2017) and vaccinia virus (Labib et al., 2012). Karash et al. (2016) designed an analogous aptasensor to detect H5N1 in chicken tracheal samples. Unlike previous methods, they used network-like thiocyanic acid/AuNPs to amplify the signal. This aptasensor finished detection within 1 h with a detection limit of 1 HAU for the H5N1(+) tracheal chicken swab samples, comparable to the conventional RT-PCR method.

Giamberardino et al. (2013) fabricated an electrochemical impedance aptasensor for rapidly detecting noroviruses from clinical samples. This aptasensor employs an AuNP-modified screen-printed carbon electrode modified with an aptamer. The working mechanism was similar to the sensors introduced above. The detecting signal was a decreased redox current, measured by square wave voltammetry. The detection limit of this aptasensor was 180 virus particles. Although the aptasensor showed promise in on-site application, it needs further development before clinical applications would be feasible.

##### Aptasensors with enzymes

The basic mechanism of aptasensors with enzymes is shown in Figure 3B. Electrochemical impedance spectroscopy (EIS) is an electrochemical measuring method that applies a small amplitude sinusoidal potential or current as a disturbance signal. In EIS bio-sensors, the incident sinusoidal wave changes when it passes through the electrode, and these changes reflect the characteristics of the electrode. Bai et al. (2018) modified the gold working electrode with aptamers to build an EIS biosensor for detecting H1N1 virus. The detection limit was 0.9 pg/μL. Based on an increased ion strength, Fu et al. (2014) proposed an aptasensor for detecting H5N1. In this aptasensor, the capturing aptamer was fixed on magnetic beads, and AuNPs-GOx-ConA complexes were used to trigger an enzyme catalysis reaction, which increased the ionic strength and decreased the impedance. The change in impedance was detected by EIS. This aptasensor detected H5N1 as low as 8 × 10^−4^ HAU in 200μL samples. The EIS strategy was also adopted in aptasensors for detecting HCV (Ghanbari et al., 2017), in which a glassy carbon electrode was modified with graphene quantum dots. The capturing aptamer, specific to the HCV core antigen, was immobilized onto the glassy carbon electrodes by the noncovalent electrostatic interactions, hydrogen-bonding and π-π stacking. The ferricyanide/ferrocyanide was employed as the signal reporter. This compound slightly inhibited electron transfer caused by redox. Once the HCV core antigen combined with the aptamer, the complex strengthened the inhibition. These changes were measured by EIS. This aptasensor detected 3.3 pg/ml, with two different linearity ranges, 10–70 pg/ml and 70–400 pg/ml, much more effective than other reported PCR or EIS methods.

Nanogates are nanodevices that control chemical or biological reactions. The basic mechanism is shown in Figure 3C. Utilizing this technology, Wang et al. (2015) developed a label-free aptasensor for AIV H5N1. To fabricate the bio-nanogate, two thiolated ssDNA probes hybridized with the aptamer and were fixed on a nanoporous gold film. Then, this “gate” covered the surface of a lactate dehydrogenase-coated glassy carbon electrode. The hybridization of ssDNA and aptamer restricted the enzymatic reaction by keeping lactate dehydrogenase from its coenzymes and substrates in the testing solution. The binding of the virus to aptamers caused the aptamers to dissociate from the ssDNAs, “opening” the gate and allowing lactate dehydrogenase to contact the testing solution and react with its coenzymes and substrates. The enzymatic reaction produced a current signal on the electrode. This biosensor detected H5N1 as low as 2^−9^ HAU in about 1 h and had a linear range of 2^−10^-2^2^ HAU.

Another electrochemical aptasensor, based on an enzymatic reaction for detecting H5N1, involves the AuNP-modified electrode functionalized with 3-mercaptopropionic acid and coated with a DNA aptamer to recognize the targets (Diba et al., 2015). An anti-H5N1 antibody, modified with alkaline phosphatase, generated an electrocatalytic reaction with the substrates. The lowest detectable concentration of this biosensor was 100 fM, and the linear range was 100 fM to 10 pM.

##### FET aptasensors

FET is a type of voltage-controlled semiconductor device that regulates electrical behaviors with an electric field. FET aptasensors immobilize aptamers on FET to detect changes in the charge distribution as a result of the binding of aptamers and target molecules. A diamond-aptamer FET sensor was investigated for detecting HIV-1 (Rahim Ruslinda et al., 2013). The mechanism of this diamond-FET aptasensor is shown in Figure 3D. In this sensor, the aptamer RNA^Tat^, against the Tat protein of HIV-1, was linked to an aminated diamond surface by terephthalic acid. When virus was captured by the RNA^Tat^ aptamer, a second strand, which could also bind the Tat protein, was added to change the potential gate voltage by transforming the duplex structure of itself and of the initial aptamer. The change in the gate potential reflected the binding of aptamer and analyte. This aptasensor detected 1 nM HIV-1 Tat protein in samples.

#### Piezoelectric Transducers

Piezoelectric effect is the ability of certain materials to generate an electric charge in response to applied mechanical stress. Quartz crystal microbalance (QCM), a type of piezoelectric transducer, uses the piezoelectric properties of quartz crystals to translate changes on the quartz crystal electrode surface into changes in the output signal frequency. In QCM aptasensors, the aptamer is fixed on the quartz crystal electrode to capture the target, and the combination of the aptamer and target changes the quality of the pole, which is then transduced into detectable frequency changes. Minunni et al. (2004) used an RNA aptamer to develop a biosensor for detecting the Tat protein of HIV-1. The lowest detectable concentration was 0.25 mg/L, and the sensor was regenerated with NaOH and alcohol. Comparing an antibody-based sensor with this aptasensor, the antibody-based sensor had a wider linear range but a lower sensitivity. This method was reproducible. Wang and Li (2013) employed an ssDNA crosslinked polymeric hydrogel in a QCM aptasensor for rapid and accurate detection of AIV H5N1. The aptamer, specific to the surface protein of H5N1, was hybridized with an ssDNA and crosslinked with the polymer hydrogel, a network of water-insoluble polymer chains. The aptamer-ssDNA gel was fixed on a gold surface using a self-assembled monolayer method. In the absence of target virus, the gel retained a shrunken state. The combination of aptamer and the virus disrupted the connection between the aptamer and the ssDNA, causing the gel to swell. These changes were transduced to a detectable decreased frequency. The detection process took 30 min, with a detection limit of 0.0128 HAU. Compared with the antibody-based QCM sensor, the aptasensor had an improved detection time and detection limit.

Atomic force microscopy is a type of scanning probe microscopy with excellent resolution. This technology works by controlling and detecting the interactions between the sample and the mechanical probe. In a study by Pleshakova et al. (2018), an aptamer specific to the HCV core antigen was immobilized on an atomic force microscopy chip, and after incubation with the antigen, the chip underwent atomic force microscopy scanning for mass spectral analysis. The detection limit was as low as 0.1 pM.

#### Other Electrical Aptasensors

A single-molecule real-time aptasensor for detecting HIV-1 was introduced by Niedzwiecki et al. (2013). This study used nanopores, the resistive-pulse technique, and an RNA aptamer with specificity to the HIV-1 nucleocapsid protein 7 called SL3. A voltage was applied across a silicon nitride membrane, and the ionic current passing through the nanopores on the membrane was tested. When the aptamer-protein complex passed through the membrane, the current was interrupted and was replaced by a translocation event signal.

### Other

A direct virus detection method was introduced by Le et al. (2014). According to this study, RNA aptamer-modified AuNPs coated a viral envelope to form a gold nanoshell, which was visualized using transmission electron microscopy. This aptamer-based method successfully detected influenza H3N2 viral particles. Aptasensors applied in virus detection are summarized in Table 2.

**Table 2:** Summary of aptasensors applied in virus detection.

**Virus**	**Target site**	**Detection technique**	**Detection limit**	**Aptamer sequence**	**References**
H1N1	HA	Impedance aptasensor	-	-	Kirkegaard and Rozlosnik, 2017
	-	Fluorescence method	3.45 nM	5′-ACACAAATCCTATTGACCGCTGTGTGACGCAACACTCAAT-3′	Zhang et al., 2013
	Influenza A virus genes	Fluorescence method	-	5′-CCCTTTAACCCCTTCTTCATCGAGAGTGTAGTCGGAAGAA-3′	Liu et al., 2017
	HA	Electrochemical method	10^3^ pfu/ml	5′-AATTAACCCTCACTAAAGGGCTGAGTCTCAAAACCGCAATACACTGGTTGTATGGTCGAATAAGTTAA-3′	Kiilerich-Pedersen et al., 2013
	-	EIS method	0.9pg/μL	-	Bai et al., 2018
H3N2	Surface protein	Colorimetry	11.16 μg/ml	5′-AATTAACCCATCACTAAAGGGCTGAGTCTCAAAACCGCAATAACTGGTTGTATGGTCGAATAAGTTAA-3′	Chen et al., 2016
H5N1	HA	SPR	0.128 HAU	5′-GTGTGCATGGATAGCACGTAACGGTGTAGTAGATACGTGCGGGTAGGAAGAAAGGGAAATAGTTGTCCTGTTG-3′	Bai et al., 2012
	HA	Impedance method	0.0128 HAU	5′-GTGTGCATGGATAGCACGTAACGGTGTAGTAGATACGTGCGGGTAGGAAGAAAGGGAAATAGTTGTCCTGTTG-3′	Lum et al., 2015
	HA	Impedance method	0.25 HAU for pure virus solution	5′-GTGTGCATGGATAGCACGTAACGGTGTAGTAGATACGTGCGGGTAGGAAGAAAGGGAAATAGTTGTCCTGTTG-3′	Karash et al., 2016
	HA	Electrochemical method	2^−9^ HAU	5′-GTGTGCATGGATAGCACGTAACGGTGTAGTAGATACGTGCGGGTAGGAAGAAAGGGAAATAGTTGTCCTGTTG-3′	Wang et al., 2015
	HA	Electrochemical method	100 fM	5′-TTGGGGTTATTTTGGGAGGGCGGGGGTT-3	Diba et al., 2015
	HA	QCM	0.0128 HAU	5′-GTGTGCATGGATAGCACGTAACGGTGTAGTAGTAACGTGCGGGTAGGAAGAAAGGGAAATAGTTGTCGTGTTG-3′	Wang and Li, 2013
	HA	ELASA	0.1 μg/well	5′-GGGTTTGGGTTGGGTTGGGTTTTTGGGTTTGGGTTGGGTTGGGAAAAA-3′	Shiratori et al., 2014
	-	SPR	200 EID_50_/ml	5′-CGTACGGTCGACGCTAGCCGAAGGTTGGAGTAGGCTAAATTGGGTGTGCACGTGGAGCTCGGATCC-3′	Nguyen et al., 2016
	HA	MEF	2 ng/ml in aqueous solution; 3 ng/ml in human serum	5′-TTGGGGGCGGGAGGGTTTATTGGGGTT-3′	Pang et al., 2015
	HA	Impedance aptasensor	0.0008 HAU in 200 μL sample	5′-GTGTGCATGGATAGCACGTAACGGTGTAGTAGTAACGTGCGGGTAGGAAGAAAGGGAAATAGTTGTCGTGTTG-3′	Fu et al., 2014
H9N2	-	PCR	10^2^ TCID_50_/ml	5′-CCTTGTTCTATTGAACCTCTTAGTCTGGTCCTCAGTTGGG-3′	Hmila et al., 2017
Influenza A viruses and influenza B viruses	Viral particles	Microfluidic system	-	5′-ACAGCACCACAGACCACCCGCGGATGCCGGTCCCTACGCGTCGCTGTCACGCTGGCTGTTTGTCTTCCTGCC-3′	Wang et al., 2016
Influenza virus	HA	TEM	3 × 10^8^ viral particles	-	Le et al., 2014
	Nucleoprotein	SERS	-	5′-TACgACTCACTATAgggATCCTgTATATATTTTgCAACTAATTgAATTCCCTTTAgTgAgggTT-3′	Nitsche et al., 2007; Negri et al., 2012
HIV-1	Tat protein	QCM	0.25 ppm	5′-ACGAAGCUUGAUCCCGUUUGCCGGUCGAUCGCUUCGA-3′	Tombelli et al., 2005
	Tat protein	SPR	0.12 ppm	5′-ACGAAGCUUGAUCCCGUUUGCCGGUCGAUCGCUUCGA-3′	Tombelli et al., 2005
	Reverse transcriptase (RT)	CE/LIF assay	50nM	5′-ATCCGCCTGATTAGCGATACTTACGTGAGCGTGCTGTCCCCTAAAGGTGATACGTCACTTGAGCAAAATCACCTGCAGGGG-3′	Pavski and Le, 2001
	RT	Radioactivity-based RT nucleotide incorporation assays	-	-	DeStefano and Alves Ferreira-Bravo, 2018
	Tat protein	Fluorescence method	-	5′-ACGAAGCUUGAUCCCGUUUGCCGGUCGAUCGCUUCGA-3′	Yamamoto et al., 2000a
	Tat protein	FET	1nM	5′-UCGGUCGAUCGCUUCAUAA-3′	Rahim Ruslinda et al., 2013
	Tat protein	QCM	0.25 ppm	5′-ACGAAGCUUGAUCCCGUUUGCCGGUCGAUCGCUUCGA-3′	Minunni et al., 2004
	NLC protein 7 (NCp7)	Nanopore and resistive-pulse technique	-	5′-GGACUAGCGGAGGCUAGUCC-3′	Niedzwiecki et al., 2013
HBV	HbsAg	Chemiluminescence	0.1 ng/ml	5′-GGGAATTCGAGCTCGGTACCCACAGCGAACAGCGGCGGACATAATAGTGCTTACTACGACCTGCAGGCATGCAAGCTTGG-3′	Xi et al., 2015
HCV	Envelope protein E2	ELASA	3.8–7.8 × 10^2^ FFU/ml	-	Park et al., 2013
	DNA	Colorimetry	11 nM	-	Liu et al., 2012
	Core antigen	Fluorescence assay	-	5′-GGGCCGTTCGAACCGAGCATGGATCGAGGATGGGAACACCCAGTAGGAGGATGGGCATGGCCGGACCCAAA-3′ATTAGCAGTGGGACAGTACTCAGGTCATCCTAGG-3′	Lee et al., 2007
	Core protein	ELASA	-	5′-ACTATACACAAAAATAACACGACCGACGAAAAAACACAACC-3′	Shi et al., 2014
	Core antigen	LFA	10 pg/ml with reader; 100 pg/ml with unaided eye	5′-GATCGAGGATGGGAACACCCAGTAGGAGGATGGGCATGGCCGGACCCAAAATTAGCAGTAAAAAAAAAAAAAAAAAA-3′	Wang et al., 2013
	NS5B protein	Octet aptasensor	700 pg/ml	5′-GGCCACAUUGUGAGGGGCUC-3′	Roh et al., 2011
	Core antigen	Electrochemical method	3.3 pg/ml	5′-ACTATACACAAAAATAACACGACCGACGAAAAAACACAACC-3′	Ghanbari et al., 2017
	Helicase	Fluorescence method	-	5′-GGGAGAGCGGAAGCGUGCUGGGCCACAUUGUGAGGGGCUCAGGUGGAUCGCAUGGCCGUGUCCAU-3′	Cho et al., 2004; Jun et al., 2010
	Core antigen	AFM-scanning	10^−14^ M	5′-ACGCTCGGATGCCACTACAGTAACACACACAACTTAAAATCATACAAAAAAGAGTAAATGCCTCATGGACGTGCTGGTGA-3′	Pleshakova et al., 2018
Norovirus GII	-	Chemiluminescence	80 ng/ml	5′-GGGGGTTTTCATCTGTGTGAAGACTATATGGCGCTCACATATTTCTTTC-3′	Kim et al., 2018
Norovirus GII.3	Capsids protein	Electrochemical method	180 virus particles	5′-GCTAGCGAATTCCGTACGAAGGGCGAATTCCACATTGGGCTGCAGCCCGGGGGATCC-3′	Giamberardino et al., 2013
Norovirus GII.4	P particles	In situ capture RT-qPCR assay	-	5′-CGATCAAACGTTCAAGCGGGGCCCGGAGGCGTGACTTGGACAGGCAGGCGTTACGATGCATCCCGCAAATGACGCATGA-3′	Liu et al., 2019
Dengue virus	EcoRI	Fluorescence method	-	5′-CCGACGAGCAAGTAGCTCCAAGACGAGTTCAACCCCAGAATCAGGTCGG-3′	Fletcher et al., 2010
SARS coronavirus	N protein	Chemiluminescence	2 pg/ml	5′-GGGAGAGCGGAAGCGUGCUGGGCCUGUCGUUCGCUGUCUUGCUACGUUACGUUACACGGUUGGCAUAACCCAGAGGUCGAUGG-3′	Ahn et al., 2009
Bovine viral diarrhea virus type 1	-	SPR	500 TCID_50_/ml	5′-CGTACGGAATTCGCTAGCTGCGCATCCACAAATGTATTGTCGGGGGATGGATCCGAGCTCCACGTG-3′	Park et al., 2014
Vaccinia	Intact virus particles	Impedimetric method	60 virions/ml	5′-CTCCTCTGACTGTAACCACGCGCGCCCCCGCTGTTCGAGCCGATAGAGGGCTAGTGTCATGCATAGGTAGTCCAGAAGCC-3′	Labib et al., 2012
	HA	Fluorescence method	-	5′-ATCCAGAGTGACGCAGCACGAGCCAGACATCTCACACCTGTTGCATATACATTTTGCATGGACACGGTGGCTTAGT-3′	Parekh et al., 2010
Prion	Cellular prion protein	Fluorescence method	0.3 mg/ml	-	Xiao et al., 2009
Zika virus	NS1 protein	ELISA	ng/ml in solution; 10 ng/ml in serum	5′-GATAGAATTCGAGCTCGGGCACTAGGTTGCAGGGGACTGCTCGGGATTGCGGATCAACCTAGTTGCTTCTCTCGTATGATGCGGGTCGACAAGCTTTAAT-3′	Lee and Zeng, 2017
RSV	Glycoprotein	Fluorescence polarization measurement	-	5′-TAGGGAAGAGAAGGACATATGATAGTGCGGTGAGCCGTCGGACATACAAATACTTGACTAGTACATGACCACTTGA-3′	Szakács et al., 2018

*-, no data*.

## Aptamers in Antiviral Therapy

Viral infection is an intractable problem for human health, which has been highlighted in recent years. Efficient and early treatment improves the prognosis, but current treatment of viral infections is not satisfactory. Many antiviral drugs and vaccines are inefficient due to frequent virus mutations and viruses escaping the host immune system (Dunning et al., 2014; Marascio et al., 2014; Sahu, 2015). Moreover, many antiviral drugs have strong side effects, such as rashes, central nervous system disorders, influenza-like symptoms, hematologic abnormalities, or organ damage (Vcev, 2009; Frasca et al., 2012). At the same time, antiviral drugs may interact with other drugs, leading to even lower efficacy (Soriano et al., 2015).

Viral infection involves adsorption, penetration, uncoating, synthesis of macromolecule, assembly, and release. These processes may be inhibited by using specific molecules that target virus-infected cells or virus components. As a novel targeted molecule, aptamers could be applied to antiviral therapy. Several mechanisms such as clathrin- and caveolae-mediated endocytosis, macropinocytosis and phagocytosis could aid in aptamer uptake. Aptamers are distributed to subcellular compartments by endocytic vesicles according to the physiology of the host cells (Yoon and Rossi, 2018). In the following sections, we introduce antiviral aptamers that employ various mechanisms.

### Suppressing Virus Attachment to Host Cells

Aptamers can impede virus entry into cells by affecting the virus or cell-surface receptors. The cellular protein nucleolin is thought to be involved in the attachment or entry of different viruses (Hovanessian, 2006; Xiao et al., 2011; Thongtan et al., 2012). Nucleolin interacts with the dengue virus capsid protein, taking part in the formation of infectious virus particles. This interaction was disturbed by the RNA aptamer AS1411, which bound to nucleolin (Balinsky et al., 2013). The influenza virus surface glycoprotein HA attaches to the sialic acid receptor of the host cell, playing a significant part in an early step of influenza infection (Skehel and Wiley, 2000; Eckert and Kim, 2001). An RNA aptamer, HA12-16, obstructed influenza virus infection in vulnerable cells by disabling the receptor-binding domain of the HA protein and enhancing cell viability (Kwon et al., 2014). A modified DNA aptamer, C7-35M, directly targeted the globular region of the AIV H9-type HA protein, suppressing virus attaching to host cells (Choi et al., 2011). In the penetration process of herpes simplex virus, the gD protein plays a key role by recognizing two protein receptors on target cells, herpes virus entry mediator and nectin-1 (Carfí et al., 2001). Based on this theory, two anti-herpes simplex virus-1 RNA aptamers were selected, which disturbed the interaction of the gD protein and the herpes virus entry mediator. This interference was dose-dependent (Gopinath et al., 2012). Similarly, another DNA aptamer targeting gD was selected for curbing herpes simplex virus-1 infection (Yadavalli et al., 2017).

### Inhibiting Replication of Viruses

Various enzymes play different and significant roles in the virus replication cycle. Enzymes or their corresponding substrates could be targeted by antiviral aptamers. Inhibiting the replication of viral nucleic acid is another method in antiviral therapy.

In Japanese encephalitis virus, a single methyltransferase domain catalyzes the methylation of the RNA cap in the cytoplasm. This domain is on the N-terminal region of the viral non-structural protein NS5. A 24-mer truncated RNA aptamer modified with 2'-O-methyl pyrimidines against the Japanese encephalitis virus methyltransferase restrained viral production in host cells (Han and Lee, 2017). Jung et al. (2018) reported an analogous study in dengue virus.

The HCV non-structural 5B polymerase is an important RNA-dependent RNA polymerase that catalyzes HCV RNA replication (Luo et al., 2000; Cheney et al., 2002). Biroccio et al. (2002) selected an RNA aptamer B.2., characterized by a stem-loop structure, that potently inhibited the non-structural 5B polymerase. The B.2. aptamer and the template RNA have different binding domains on the RNA-dependent RNA polymerase, and B.2. could noncompetitively bind the RNA polymerase, weakening its activity. Two RNA aptamers, 27v and 127v, specific to the non-structural 5B polymerase, inhibited HCV polymerase activity *in vitro* (Bellecave et al., 2008). By competing for the binding sites of the polymerase with viral RNA template, the aptamer 27v blocked both the initiation and the elongation of viral RNA synthesis, while the aptamer 127v inhibited the initiation and postinitiation events.

The multifunctional regulatory protein pUL84 is fundamental in the early phase of human cytomegalovirus replication. By mediating the cellular importin-α/β pathway, nuclear localization signal is involved in the nuclear trafficking of pUL84. In Kaiser's research, peptide aptamers aimed at the nuclear localization signal domain of pUL84 abrogated the nuclear translocation of this viral replication factor by restraining the interaction between importin-α proteins and pUL84 (Kaiser et al., 2009).

The HBV core protein is significant in the production of the HBV nucleocapsid and affects viral envelopment (Deres et al., 2003; Roseman et al., 2005). Aptamer No.28 efficiently impeded HBV nucleocapsid assembly and suppressed viral replication (Zhang et al., 2014). Similarly, under intracellular conditions, a peptide aptamer against the HBV core protein prevented viral replication by disturbing capsid formation (Butz et al., 2001).

The HIV-1 nucleocapsid protein is crucial in the encapsidation of virus nucleic acids and the installment of virus particles (Kim et al., 2002). The retroviral psi packaging element is a cis-acting RNA element in the genome of HIV and is involved with regulating the packaging process of the viral genome in replication (Lever et al., 1989; McBride and Panganiban, 1997; McBride et al., 1997; Lever, 2007). Based on this, RNA aptamers specific to the HIV nucleocapsid protein were selected for disturbing viral packaging. The aptamers worked by competing for the psi RNA (Kim et al., 2002).

### As a Delivery Tool

Small interfering RNA (siRNA) is a category of dsRNA 20-25 base pairs in size. By inducing degradation of mRNA after transcription, siRNA can inhibit the expression and translation of corresponding genes (Agrawal et al., 2003). SiRNA can also interfere with the formation of the chromatin structure of a genome (Hamilton and Baulcombe, 1999; Elbashir et al., 2001). SiRNA has shown great value in biomedical research and drug development since its discovery. However, off-targeting restricts siRNA applications in therapies (Shen et al., 2012). Aptamers are a desirable siRNA delivery tool of siRNA due to their high specificity, affinity to targets, and low toxicity.

The application of aptamer-siRNA in HIV-1 therapy has been a hot topic in recent years. Envelope glycoprotein GP120 (gp120) is a glycoprotein expressed on the HIV envelope. By attaching to the specific cell surface receptors, gp120 participates in the process of virus entry into cells (Dalgleish et al., 1984; Curtis et al., 1992). Zhou and coworkers employed the anti-gp120 aptamer-siRNA chimera for HIV-1 treatment. The aptamer carried the siRNA to cells infected with HIV-1, then the siRNA inhibited HIV replication (Zhou et al., 2008). In later studies, Zhou found that the aptamer could neutralize virus infection and transfer functional siRNAs to HIV-1 infected cells (Zhou et al., 2011). To improve the transport capacity of aptamers as siRNA carriers, researchers modified gp120-specific aptamers with a 3′ 7-carbon linker, which was bound with a 16-nucleotide 2′ OMe/2′ Fl GC-rich bridge sequence. The sequence promoted the non-covalent combination and interaction of various siRNAs with the aptamers (Zhou et al., 2013). The aptamer-siRNA system has also been studied by other researchers (Catuogno et al., 2015).

### Others

To mitigate HIV-associated cardiomyopathy, Lopes de Campos et al. (2014) employed an anti-gp120 aptamer UCLA1. By directly binding to HIV-1 and neutralizing the virus, the aptamer protected cardiomyocytes from apoptosis and indirectly prevented infection of monocyte-derived macrophages.

Aptamers applied in antiviral therapy are summarized in Table 3.

**Table 3:** Summary of aptamers and aptamer-based experiments in antiviral therapy.

**Virus**	**Aptamer type**	**Application**	**Aptamer sequence**	**References**
DENV	RNA	Blocking the interaction between NCL and DENV capsid protein	5′-GGTGGTGGTGGTTGTGGTGGTGGTGG-3′	Balinsky et al., 2013
	RNA	Binding and inhibiting the methylation activity of MTase	5′-GGGAGAGCGGAAGCGUGCUGGGCCCAGUGGUUGGGCACAUAUAGACUGUGUAAUUCGUAUAGUGUGCAUAACCCAGAGGUCGAUGGAUCCCC-3′	Jung et al., 2018
Influenza virus	ssDNA	Targeting the HA	5′-AACGCTCACTCCCCCAAGAAGAACCCCCCCCCCCCCCCCCCCCCCAGTGAGCGTT-3′	Musafia et al., 2014
Influenza virus (H5N1)	DNA	Binding to the HA1 protein to disrupt virus entry	5′-GAATTCAGTCGGACAGCGGGGTTCCCATGCGGATGTTATAAAGCAGTCGCTTATAAGGGATGGACGAATATCGTCTCCC-3′	Cheng et al., 2008
Influenza A virus	DNA	Binding and inhibiting the endonuclease	5′-CCGTAATACGACTCACTATAGGGGAGCTCGGTACCGAATTCGCAAGCGTCTGCATCCCGGTGGGACCATTAAAGCTTTGCAGAGAGGATCCTT-3′	Yuan et al., 2015
AIV	DNA	Hindering viral absorption or inhibiting HA-mediated membrane fusion by binding to HA	5′-GCTGCAATACTCATGGACAGCCTCCTGGGGTCAGGCTCAGACATTGATAAAGCGACATCGGTCTGGAGTACGACCCTGAA-3′ or 5′-GCTGCAATACTCATGGACAGGGGCCGCGCCTGGTCGGTTGGGTGGGTGGCGCCCGGGACGGTCTGGAGTACGACCCTGAA-3′	Zhang et al., 2015
	DNA	Recognize the HA protein and inhibit the binding of the virus	5′-ATTAACCCTCACTAAAGGGAGGTAGTTATAGTATATGGAAGGGGGTGTTATGGTCGAATAAGTTAACG-3′	Jeon et al., 2004; Choi et al., 2011
	RNA	Neutralizing the receptor-binding domain of HA	5′-GCUUGACGGAGAUCAAGGGCGAGUCUCAUACCAAGUUGAUGGGG-3′	Kwon et al., 2014
HSV-1	RNA	Binding to the gD protein to interfere with the binding of gD and the host receptors	5′-GGGAGCUCAGCCUUCACUGCACGAGAGAGGUCGUCCCCAGGGGAGAACUCGUGCUCCUGGAGGCAAGUUGACUGCUCGCUCUCAGCUGGUCAAGGGCACCACGGUCGGAUCCUG-3′	Gopinath et al., 2012
	DNA	Binding to the gD protein to interfere with the binding of gD and the host receptors	5′-GGGCACGAGAGAGGTCGTCCCCAGGGGAGAACTCGTGCTCCTGG-3′	Yadavalli et al., 2017
JEV	RNA	Suppressing JEV MTase to inhibit viral cap methylation	5′-CCACGACAGCAUGCCAAUAGAUGCGCAUGGAGACGACAGCAU-3′	Han and Lee, 2017
HBV	DNA	Targeting the HBV core protein to reduce the synthesis of extracellular HBV DNA	5′-ACGCTCGGATGCCACTACAGCTTCCCCTAATCTGGCGCTCTCATCTAATTTCCCTTCCTGCTCATGGACGTGCTGGTGAC-3′	Zhang et al., 2014
	RNA	Interfering with viral P-ε complex formation	5′-UGUUCAUGUCCUACUGUUCAAACAAAAAAACUGUGCACAAAAAUAAAUUGGGGCAUGGACA-3′	Feng et al., 2011
	DNA	Impairing virion formation by inhibiting the matrix domain- matrix binding domain interaction	5′-gcgggtcgacgtttgCACACGCGAGCCGCCATGTCTGGGCcacatccatgggcgg-3′	Orabi et al., 2015
	Recombinant proteins	Working on the core protein to disturb viral capsid formation and DNA replication	-	Butz et al., 2001; Zhang et al., 2009
	Recombinant proteins	Redistributing intracellular target protein into perinuclear inclusion bodies to inhibit viral capsid formation	-	Tomai et al., 2006
HCV	RNA	Suppressing HCV NS5B replicase	5′-UUGAACGAUUGGUAGUAGAAUAUCGUCAG-3′	Lee et al., 2013
	RNA	Recognizing the GTP binding site of NS5B to suppress the activity of polymerase	5′-CGAAGCCGCUAUGGACCAGUGGCGCGGCUUCGGCCCGACGGAGUG-3′	Biroccio et al., 2002
	DNA	Binding to NS5B and inhibiting its polymerase activity	-	Bellecave et al., 2008
	DNA	Inhibiting E2 protein binding to CD81	5′-GCGGAATTCTAATACGACTCACTATAGGGAACAGTCCGAGCCGAATGAGGAATAATCTAGCT CCTTCGCTGAGGGTCAATGCGTCATAGGATCCCCC-3′	Chen et al., 2009
Cytomegalovirus	Recombinant proteins	Neutralizing the NLS sites of pUL84 to interfere viral replication and production	-	Kaiser et al., 2009
Rabies virus	ssDNA	Recognizing the RABV glycoprotein on infected cells to inhibit the earliest stages of infection	5′-TATTTTTATATTTGTTTGACAGTCGCTTGCTTGTGTAGGCGTT-3′	Liang et al., 2014
HIV-1	RNA	Working on the nucleocapsid protein	-	Kim et al., 2002
	RNA	Preventing the gp120 from interacting with the chemokine receptor	-	Lopes de Campos et al., 2014
	RNA	-	5′-UAAUACGACUCACUAUAGGGAGACAAGACUAGACGCUCAACAGGACCGAGAGAUGCAAC UAGUGAUUUCCCUCAUAAUCAUUCUAAGAGCUUCGACAUGAGA CUCACAACAGUUCCCUUUAGUGAGGGUUAAUU-3′	London et al., 2015
	DNA	Inhibiting the RT	5′-cgcctgattagcgatactCAGGCGTTGGGGGGGGGGGG-3′ or 5′-atccgcctgattagcgatatCAGAAGGATAAACTGTCCAGAAC-3′	Ditzler et al., 2011
	RNA	Inhibiting the RT	5′-GACAGGGCCCGTTTTCCAGTGTTTTCCCCTTTATCTCCTGGGTTCGTAGGGAATTCAG-3′	Lange and Burke, 2014
	DNA	Inhibiting the RT	5′-GGGGGTGGGAGGAGGGTAGGCCTTAGGTTTCTGA-3′	Shiang et al., 2013
	DNA	Inhibiting both HIV infection and HIV-1 integrase	-	Magbanua et al., 2013
	RNA	Inhibiting RT activity by competing with the primer/template for access to RT	5′-GGGCAACCGGUGUCUACCGGGCUUCGGCCCGGUUCAAGGACACCGCCACUGC-3′	Whatley et al., 2013
HIV	DNA	Targeting delivery of siRNAs	5′-GTGACGTCCTGATCGATTGTCGCATTCGGTGTGACGATCTGCUCUAUUAGAUACAGGAGtt-3′	Zhu et al., 2012
	DNA	Inhibiting both HIV infection and HIV-1 integrase	-	Magbanua et al., 2013
	RNA	Binding to CycT1 to restrict the production of transcription elongation factor B	5′-GGTAATACGACTCACTATAGGGAGATACCAGCTTATTCAATTCCUACCAA AUACGAGCCCAUCGUCACGUUCUCUUAUCUACAGATAGTAAGTGCAATCT-3′	Um et al., 2012
Ebola virus	RNA	Depressing the activity of the polymerase by interfering with the binding of gD and the host receptors	5′-GGGAGACAAGAAUAAACGCUCAAGGCAUUUCUGCUAGUCUGGUUGUAA GAUAUUCAACACGUGAGUUUCGACAGGAGGCUCACAACAGGC-3′ or 5′-GGGAGACAAGAAUAAACGCUCAACGUUCAGUAUAACAGUCCGAGUCUA ACACACAAUGGGACACUGAAUUCGACAGGAGGCUCACAACAGGC-3′	Binning et al., 2013
HPV	RNA	Binding to virus capsid and interrupting the binding of the virus capsid to heparan sulfate (HS) receptors	5′-GGGAACAAAAGCUGCACAGGUUACCCCCGCUUGGGUCUCCCUAUAGUGAGUCGUAUUA-3′ or 5′-GGGAACAAAAGCUGCACAGGUUACCCCCGCUUGGGUCUCC-3′	Valencia-Reséndiz et al., 2018

## Virus-Targeting Aptamers

In addition to the aptamers mentioned above, there are many aptamers for detecting different viruses that have not been used in virus detection or antiviral therapy. Table 4 summarizes aptamers that target different viruses.

**Table 4:** Summary of aptamers that have not been used in virus detection or antiviral therapy.

**Virus**	**Binding site**	**Sequence**	**References**
HPV-16	L1 protein	5′-GGGAACAAAAGCUGCACAGGUUACCCCCGCUUGGGUCUCCCUAUAGU GAGUCGUAUUA-3′	Leija-Montoya et al., 2014
HBV	HBsAg	5′-GTTGATTGCGTGTCAATCATGGCCGTCTATAATGATCG TAAACGACGGGTCATGTGTATGTTGGGGATTGGGACCTGATTGAGTTCAGCCCACATAC-3′	Liu et al., 2010
HCV	Envelope glycoprotein E2	5′-GAATGAGGAATAATCTAGCTCCTTCGCTGA-3′	Chen et al., 2009
Human noroviruses (GII.2 and GII.4)	Capsid protein	5′-GTCTGTAGTAGGGAGGATGGTCCGGGGCCCCGAGACGACGTTATCAGGC-3′	Beier et al., 2014
Dengue virus	Envelop protein domain III	5′-GCACCGGGCAGGACGTCCGGGGTCCTCGGGGGGC-3′	Chen et al., 2015
	Envelop protein domain III	5′-CGGCATTCTCCTGCTACGAGG-CGCTGCGGTACACCCCGACTCCAC – GAGCCACTGTCTACGGACATCTG-3′	Gandham et al., 2014
SARS CoV	Nucleocapsid protein	5′-GCAATGGTACGGTACTTCCGGATGCGGAAACTGGCTAATTGGTGAGGC TGGGGCGGTCGTGCAGCAAAAGTGCACGCTACTTTGCTAA-3	Cho et al., 2011
Vaccinia	Surface protein	5′-ATCGTCTGCTCCGTCCAATAGTGCATTGAAACTTCTGCATCCTCGTTTGGT GTGAGGTCGTGC-3′	Tang et al., 2009
Ebolavirus	Soluble glycoprotein	5′-GGGCGCUCAAUUUUUUAUUGCAUUUUUCUUUGAGCGCCC-3′	Shubham et al., 2018

## Conclusions and Future Perspectives

Aptamer technologies are being increasingly applied in research, for diagnosis and therapy, because of the high binding specificity and affinity, and other advantages of aptamers (Table 1). Although many studies have been published on virus detection and treatment, few aptamer-based products are commercially available for clinical diagnosis and therapy (González et al., 2016). In addition, several of these studies have compared the aptasensor with other detection methods in detail (Minunni et al., 2004; Wang and Li, 2013; Ghanbari et al., 2017). Nevertheless, aptamer technologies still face many impediments; for instance, the aptamer screening process is difficult. Even though the principles of SELEX are the same for diverse targets, the experimental details are often quite different, requiring significant time and effort to establish suitable reaction conditions. Also, selection failure is common due to significant uncertainties in PCR bias, PCR artifacts, and background binders (Rozenblum et al., 2016). Another difficulty in developing aptamer technologies is that aptamers are screened under certain conditions which do not always exactly replicate the conditions of complex clinical samples, so the structure, function, the binding affinity and specificity of aptamer could possibly be changed in clinical samples. Another hurdle for aptamer technologies is that special bases are used to construct aptamers to optimize their affinity and specificity, causing increased synthesis costs. To optimize the selection process and aptamer properties, researchers have proposed improved strategies, such as SOMAmer, bead-based selection, Cell-SELEX and microfluidics technology, and have achieved remarkable results (Sun and Zu, 2015). In addition, diverse chemical modifications to the nucleotide composition of aptamers, including pegylation, have improved the metabolic stability of aptamers. Aptamer applications in virus detection and therapies can be improved by (1) improving aptamer screening technologies; (2) further understanding the 3D models and the factors influencing the binding of aptamers and their targets; and (3) further verifying aptamers as diagnostic and therapeutic agents both *in vitro* and *in vivo*. In conclusion, while there are still some gaps in developing aptamers for clinical applications, aptamers will be widely used in virus detection and therapy with the improvement of the relevant technologies.

## Author Contributions

XZ, JW, JG, and LS conceived the work and discussed the content. XZ drafted the manuscript. JW, JG, and LS were responsible for revising it. LM critically reviewed, edited, and finalized the manuscript for submission.

### Conflict of Interest Statement

The authors declare that the research was conducted in the absence of any commercial or financial relationships that could be construed as a potential conflict of interest.
